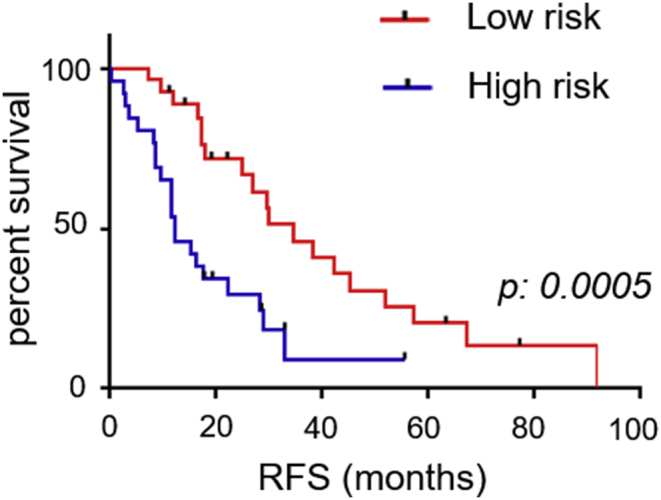# Characterisation of the immune-related transcriptome in resected biliary tract cancers

**DOI:** 10.1016/j.ejca.2017.09.005

**Published:** 2017-11

**Authors:** Michele Ghidini, Luciano Cascione, Pietro Carotenuto, Andrea Lampis, Francesco Trevisani, Maria Chiara Previdi, Jens C. Hahne, Ian Said-Huntingford, Maya Raj, Alessandro Zerbi, Claudia Mescoli, Umberto Cillo, Massimo Rugge, Massimo Roncalli, Guido Torzilli, Lorenza Rimassa, Armando Santoro, Nicola Valeri, Matteo Fassan, Chiara Braconi

**Affiliations:** a The Institute of Cancer Research, Cotswold Road, London, SM2 5NG, UK; b Humanitas Cancer Center, Humanitas Clinical and Research Center, Via Manzoni, 113, Rozzano, Milan, 20089, Italy; c ASST Hospital of Cremona, Viale Concordia, 1, Cremona, 26100, Italy; d Institute of Oncology Research, Via Vela 6, Bellinzona, 6500, Switzerland; e San Raffaele Scientific Institute, Via Olgettina, Milan, 20132, Italy; f University of Padua, Via Gabelli 61, Padova, 35100, Italy; g Humanitas University, Via Manzoni, 113, Rozzano, Milan, 20089, Italy; h The Royal Marsden NHS Foundation Trust, London and Surrey, Downs Road, SM2 5PT, UK

**Keywords:** Cholangiocarcinoma, CTLA4, Treg, Adjuvant, CD80, BTC, biliary tract cancer, CCA, cholangiocarcinoma, ECC, extrahepatic CCA, ICC, intrahepatic CCA, GBC, gallbladder cancer, RFS, relapse-free survival, TT, tumour tissue, AT, adjacent tissue, FFPE, formalin fixed paraffin embedded, PBK, PDZ binding kinase, CTLA4, cytotoxic T-lymphocyte antigen-4, TGFB1, transforming growth factor beta, IL-6, interleukin 6, CD80, cluster of differentiation 80, IP, immune profile, R1, positive resection margins, R0, clear resection margins, POLR2A, RNA polymerase II subunit A, IPA, ingenuity pathway analysis, Treg, T regulatory cell, APC, antigen-presenting cell

## Abstract

Although biliary tract cancers (BTCs) are known to have an inflammatory component, a detailed characterisation of immune-related transcripts has never been performed. In these studies, nCounter PanCancer Immune Profiling Panel was used to assess the expression of 770 immune-related transcripts in the tumour tissues (TTs) and matched adjacent tissues (ATs) of resected BTCs. Cox regression analysis and Kaplan–Meier methods were used to correlate findings with relapse-free survival (RFS). The first analysis in the TT and AT of an exploratory set (n = 22) showed deregulation of 39 transcripts associated with T-cell activation. Risk of recurrence was associated with a greater number of genes deregulated in AT in comparison to TT. Analysis in the whole set (n = 53) showed a correlation between AT cytotoxic T-lymphocyte antigen-4 (CTLA4) expression and RFS, which maintained statistical significance at multivariate analysis. CTLA4 expression correlated with forkhead box P3 (FOXP3) expression, suggesting enrichment in T regulatory cells. CTLA4 is known to act by binding to the cluster of differentiation 80 (CD80). No association was seen between AT CD80 expression and RFS. However, CD80 expression differentiated prognosis in patients who received adjuvant chemotherapy. We showed that the immunomodulatory transcriptome is deregulated in resected BTCs. Our study includes a small number of patients and does not enable to draw definitive conclusions; however, it provides useful insights into potential transcripts that may deserve further investigation in larger cohorts of patients.

**Transcript Profiling:**

Nanostring data have been submitted to GEO repository: GSE90698 and GSE90699.

## Highlights

• BTCs harbour a derangement of immune-related transcripts with an important role for the cytotoxic T-lymphocyte antigen-4 (CTLA4) axis.
• CTLA4 expression in the peritumoural areas correlates with outcome and represents a potential promising prognostic factor.
• Our data suggest that immunotherapy may have an impact in BTCs as a mean to re-address the host immune response to the tumour.

## 1 Introduction

Biliary tract cancers (BTCs) arise from the epithelium of bile ducts and gallbladder [1]. Ninety percent of BTCs are adenocarcinomas, also called cholangiocarcinomas (CCAs). Although intrahepatic (ICC) and extrahepatic (ECC) CCAs harbour at least partially different molecular features, the clinical management of BTC does not differ according to the subtypes [2], [3], [4]. In phase III clinical trials, ICC, ECC and gallbladder cancers are grouped together as BTCs [5], [6].

Surgery is the only curative treatment modality in BTCs. However, the 5-year survival rate for patients with resected BTCs is only 10–50% [7], [8]. Therefore, consideration of adjuvant therapy is justified. Nevertheless, the BILCAP study suggests that adjuvant chemotherapy can improve the outcome of resected BTCs [9]. It is likely that the discovery of prognostic factors associated with the biological aggressiveness of these tumours will guide the post-surgical management of BTCs. Recent evidence suggests that the host immune response modulates the effect of chemotherapy in solid tumours [10], [11]. Inflammation and immune modulation are recognised as driving forces in the pathogenesis of BTCs [2], [12], [13], [14], [15], [16], [17], [18]. However, detailed immune profiling of tumour and peritumoural areas has not been performed in BTCs. In this study, we aimed at characterising the deregulation of immune-related transcripts in resected BTCs and exploring their potential as prognostic markers.

## 2 Patients and methods

### 2.1 Patient population

We assessed a set of 53 patients with resected BTCs treated at the Humanitas Clinical and Research Center, Rozzano (Milan, Italy) and the University Hospital of Padua (Padua, Italy). The study protocol was given ethical approval by Institutional Review Boards. All patients underwent curative surgery (Table 1). Data collection was performed retrospectively. Relapse-free survival (RFS) was used as end-point of the study. The first analysis was performed in an exploratory subset including 22 cases, for which nanostring analyses were performed in tumour (TTs) and adjacent tissues (ATs). Extended analyses were carried out in the whole set of patients (n = 53) by performing nanostring analyses in the AT only. mRNA expression profiling was analysed with a commercially available nCounter PanCancer Immune Profiling Panel from NanoString Technologies (Seattle, WA, USA), as per manufacturer's instructions.

**Table 1:** Demographics of patients.

Characteristics	Exploratory set	Entire set
	Number (%)	Number (%)
Patients	22	53
***Gender***		
Female	10 (45%)	23 (43%)
Male	12 (55%)	30 (57%)
***Age***		
Median (years) [range]	65 [41–76]	63 [38–77]
***Tumour site***		
ICC	4 (18%)	21 (40%)
ECC	10 (45%)	24 (45%)
GBC	8 (37%)	8 (15%)
***T stage***		
T1	1 (5%)	8 (15%)
T2	9 (40%)	29 (55%)
T3	7 (32%)	11 (21%)
T4	5 (23%)	5 (9%)
***N stage***		
N0	11 (50%)	24 (45%)
N1	11 (50%)	18 (34%)
Nx	0 (0%)	11 (21%)
***Resection margins***		
Negative (R0)	17 (77%)	47 (89%)
Positive (R1)	5 (23%)	6 (11%)
***Adjuvant chemotherapy***		
Yes	11 (50%)	33 (62%)
No	11 (50%)	20 (38%)
***Recurrence***		
Yes	15 (68%)	40 (75%)
No	7 (32%)	13 (25%)
***Follow-up***		
Median RFS (months) [range]	13.1 [0.33–63.3]	17.8 [0.33–91.73]

ICC, intrahepatic cholangiocarcinoma; ECC, extrahepatic cholangiocarcinoma; GBC, gallbladder cancer; RFS, relapse-free survival.

### 2.22.2 Statistical analysis

RFS was defined as the time between surgery and radiological evidence of tumour relapse. RFS was chosen as end-point of the study to avoid bias related to different treatments in the metastatic setting. Patients alive and without evidence of tumour relapse at the time of the analysis were censored at last follow-up. The Kaplan–Meier method was used to calculate survival estimates, and comparison of the treatment arms was carried out using a log-rank analysis. Hazard ratios (HRs) and 95% confidence intervals (CIs) were obtained by Cox regression. Multivariate Cox regression was used to assess whether an interaction remained significant after addition of prognostic variables including tumour site, T, N, resection margins, adjuvant treatment and institution. We correlated mRNA expression with clinical indicators dichotomising samples at the median gene expression in high- versus low-expression group. P value for statistical significance was set at <0.05. Statistical analyses were performed by R and GraphPad Prism 6 (La Jolla, CA, USA), in a blinded fashion by an external biostatistician.

Further methods can be found in the Supplementary file.

## 3 Results

**Fig. 1:**
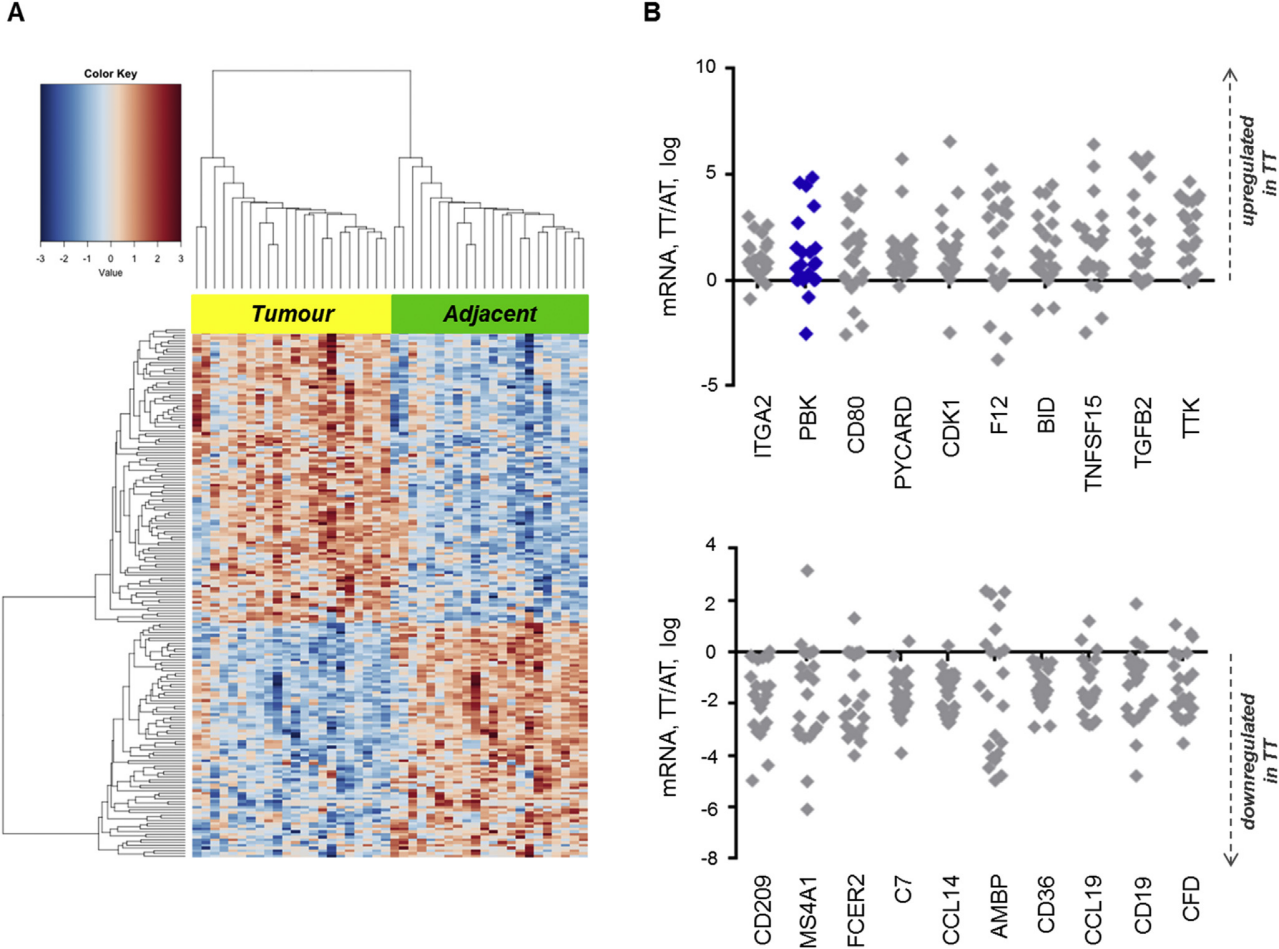
**Immunomodulatory transcripts are deregulated in biliary tract cancers (BTCs).** RNA from tumour tissue (TT) and adjacent tissue (AT) of BTCs of the exploratory set were subjected to nCounter analysis of 770 immune-related transcripts. (A) Heatmap showing mRNAs deregulated in TT compared with AT (adjusted p < 0.05). (B) Graphic representation of the top 20 transcripts deregulated >2-fold in TT versus AT (p < 0.05). Each dot represents the log of the ratio between TT and AT for each patient. Positive values indicate upregulation in TT; negative values indicate downregulation in TT.

Previous studies suggest that BTCs are inflammatory cancers associated with derangement of cytokines and recruitment of immune cells. To investigate if the transcriptomic immune profile is deregulated in BTC, we started by performing a comprehensive immune profiling of 770 immune-related transcripts in TT and AT of a set including 22 resected BTCs. Demographic characteristics of patients are listed in Table 1. One-hundred ninety-five transcripts were aberrantly expressed (109 upregulated and 86 downregulated) in TT compared with AT (p < 0.05) (Fig. 1A). Of these, 39 transcripts were deregulated >2-fold in TT compared with AT (Suppl Table 1). Ingenuity Pathway Analysis of this set of genes showed involvement in the inflammatory response, immune-cell trafficking and T-cell deregulation (Suppl Table 2). This network includes genes such as cluster of differentiation 80 (CD80), which regulates T-cell activation by binding to cytotoxic T-lymphocyte antigen-4 (CTLA4) (Suppl Fig. 1). Amongst the transcripts deregulated by an average >2-fold (Suppl Table 1, Fig. 1B), there is PDZ-binding kinase, a kinase produced by lymphokine-activated killer T-cells, which was previously found to be a CCA-specific transcript associated with prognosis [19].

It is recognised that deregulation of the immune system may be a cause of cancer progression by creating a favourable microenvironment for cancer growth and escape. Thus, we extended our analysis to genes that were not differently expressed between cancer and ATs, hypothesising that genes expressed in the tumour compartment may reflect enrichment of immune cells within the tumour content, whereas genes expressed in the adjacent compartment reflect deregulation of immune infiltrate in the normal tissue that creates a favourable soil for cancer-cell growth. We began by analysing the clinical impact of BTC type on survival. RFS was not statistically different according to tumour site (Suppl Fig. 2); thus, we grouped our tumour types together in line with the analyses performed in the major clinical trials that have generated recommendation for clinical practice [5], [9].

**Fig. 2:**
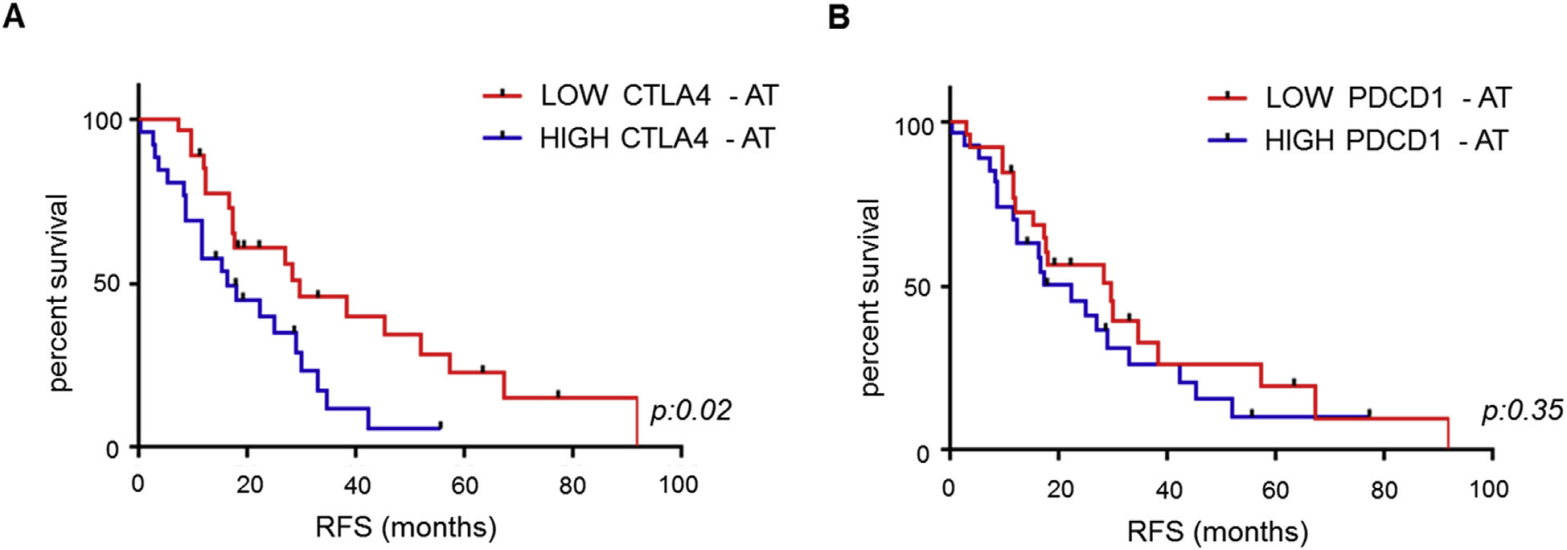
**Cytotoxic T-lymphocyte antigen-4 (CTLA4) is associated with relapse-free survival.** (A) CTLA4 mRNA was assessed by nCounter analysis in the entire set (n = 53). Cases were divided according to low and high expression of mRNA in the adjacent tissue (AT) (using median as cut-off). Median overall survival was 16.27 months in cases with high CTLA4 expression, whereas it was 29.53 months in low CTLA4 cases. (B) PDCD1 mRNA was assessed by nCounter analysis in the entire set. Cases were divided according to low and high expression of mRNA in the AT (using median as cut-off). RFS, relapse-free survival.

**Fig. 3:**
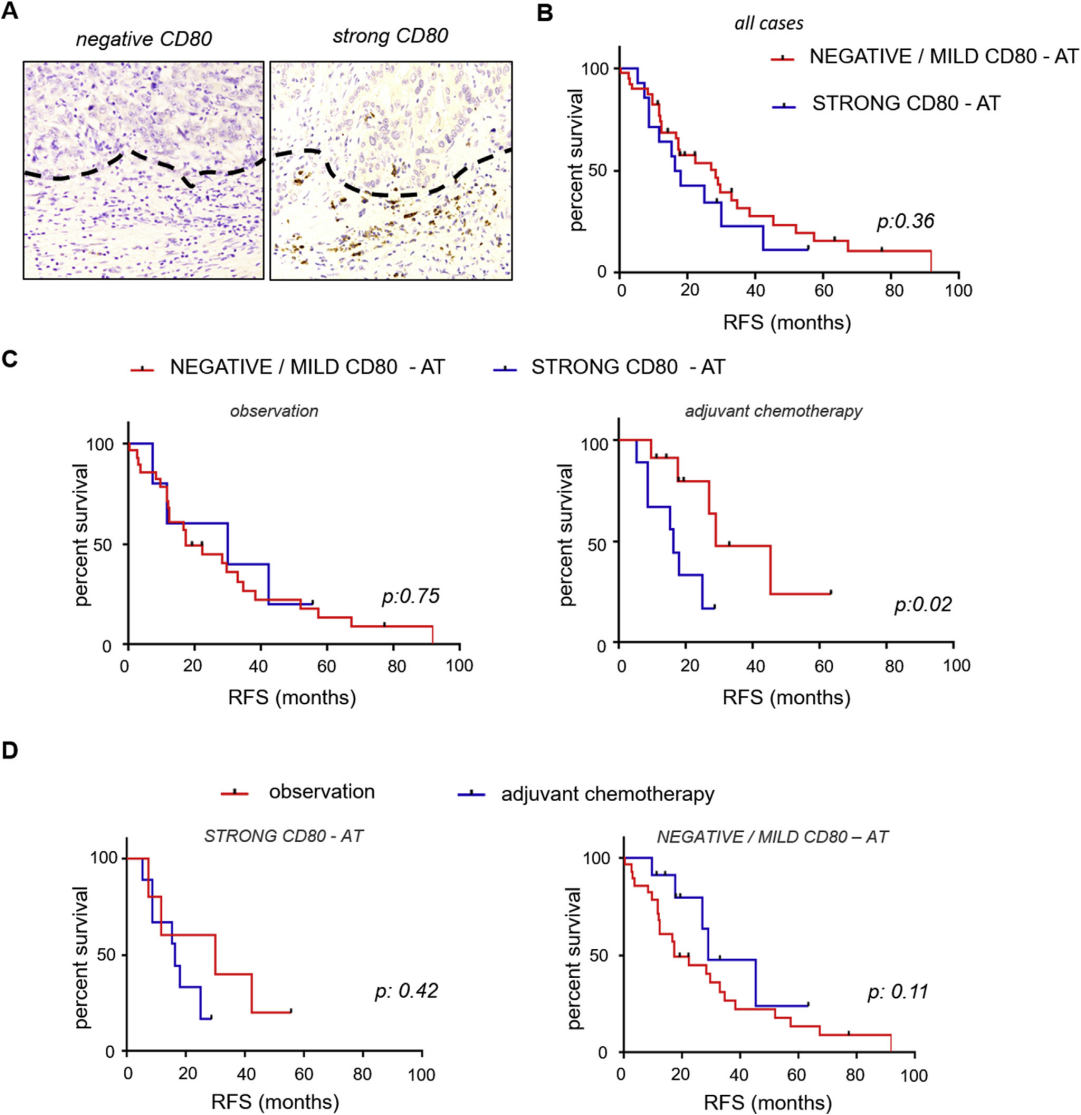
**Cluster of differentiation 80 (CD80) protein expression and prognosis.** (A) The presence of CD80-positive cells in the adjacent tissue (AT) was assessed by immunohistochemistry (IHC). Examples of CD80-poor and CD80-rich biliary tract cancer cases are shown. (B) CD80 protein expression was assessed by IHC in the AT of the entire set (n = 53). Cases were divided according to protein expression of CD80 in the AT (+++ versus −/+/++). (C) The same analysis was performed in the cohort receiving post-operative observation or adjuvant chemotherapy. In the latter median relapse-free survival (RFS) was 16.27 months in strong (+++) CD80 (n = 11) whereas it was 28.8 months in the other (−/+/++) group (n = 9). (D) CD80 expression was assessed by IHC in the AT (n = 53). In strong CD80 cases median RFS was 16.27 months in the adjuvant group compared to 29.93 months in the observation group. In the cases with negative/mild expression of CD80, adjuvant chemotherapy improved median RFS from 17.23 months to 28.8 months (non-significant).

We derived a list of genes, whose expression was associated with RFS at univariate analysis, and we shortlisted genes that maintained statistical significance at multivariate analysis (Suppl Fig. 3). Interestingly, tumours with high expression of NOTCH1 were significantly associated with lower RFS (Suppl Fig. 3) as previously described [20], [21]. We observed that risk of recurrence was associated with a greater number of genes deregulated in AT in comparison with genes altered within the TT, suggesting that the inflammatory–immunomodulatory background plays an important role in BTC development and progression (Suppl Fig. 3). As much as we do believe that deregulation of immune transcripts in the TT may deserve further investigation as prognostic markers; for the purpose of this study, we decided to focus on the transcripts deregulated in the peritumoural tissue, given this is an area of recognised activity of the immune cells that localise in the peritumoural border [22], [23]. Thus, we extended our nCounter profiling to the AT of the 53 cases of the entire set. Univariate and multivariate analyses were run to identify transcripts that were associated with RFS (Suppl Table 3). Amongst several genes, we focussed on those that represented targetable pathways. While we have not observed any correlation between PD1 expression and RFS (Fig. 2), we observed an inverse correlation between RFS and expression of CTLA4 (Fig. 2 and Suppl Fig. 4A). Therapeutics are available to target CTLA4, and therefore we focussed our attention on this transcript. The relative expression of CTLA4 did not differ across the different subtypes in the set of patients for which TT and AT expression was available (Suppl Fig. 4B and C). CTLA4 maintained its prognostic value also in the multivariate analysis (Suppl Table 4). CTLA4 is mainly displayed on activated T regulatory cells (Tregs) and is receptor to CD80 and CD86 [24]. We noticed a correlation between CTLA4 expression and FOXP3 expression, suggesting that cases with high CTLA4 indeed have an enrichment of Tregs (Suppl Fig. 4D). As CD80 and CD86 co-contribute to the activation of Treg through interaction with CTLA4, we looked at the correlation between RFS and CD80 and CD86 expression. Neither CD80 nor CD86 could differentiate prognosis when all the cases were grouped together. However, there was a trend for worse survival for high CD80-AT cases in patients undergoing adjuvant chemotherapy (Suppl Fig. 5), in line with published data on the role of Tregs in response to chemotherapy [10], [11]. To further investigate this finding, we examined the count of CD80-positive cells using immunohistochemistry (IHC). In the subgroup that received adjuvant chemotherapy, we confirmed that cases with strong AT CD80 expression had a longer median RFS (28.8 months) compared to cases with negative/mild CD80 expression (16.27 months) (HR 3.25 [95% CI: 1.31–15.54; p = 0.02]; Fig. 3A–C). As this may suggest a value of CD80 as a biomarker of sensitivity to chemotherapy, we looked at the effect of adjuvant chemotherapy in patients with strong CD80 expression compared to the negative/mild ones. While no advantage was observed for cases with strong CD80 expression, adjuvant chemotherapy tended to improve RFS in cases with negative/mild expression of CD80, even though statistical significance was not reached (Fig. 3D). We acknowledge that the low numbers in these analyses do not allow for any definitive conclusion, but we believe CD80 may be a promising marker to be studied in larger cohorts.

Given these data suggest that an immunomodulatory network plays a role in BTC relapse, we searched for a gene signature that could identify cases with higher risk of relapse. We derived a gene signature using the 42 genes that were associated to RFS (Suppl Table 3). This signature could significantly differentiate cases with higher risk of relapse (Suppl Fig. 6). When the predictive performance of the gene signature and CTLA4 alone were compared, the gene signature seemed to have only a slightly stronger predictive power (concordance probability estimate [CPE] 0.62 versus 0.59, p = 0.03; Akaike's information criterion [AIC] 244 versus 241, p = 0.03).

## 4 Discussion

BTCs are recognised to be inflammatory cancers with associated activation of the immune system [16], [18], [24], [25], [26]. Loss of immune response in BTCs is associated with lack of peritumoural inflammatory infiltrate and poorer survival [27], [28]. It is reasonable to hypothesise that the activation of immune-escaping phenomena can favour tumour relapse after surgical resection [29], [30], [31]. In this study, we characterised the immune response of resected BTCs and observed that the deregulation of immunomodulatory transcripts in peritumoural areas can create an immunosuppressive milieu that facilitates tumour relapse, likely through the activation of the CTLA4 axis. We focussed our validation on CTLA4 because it is a target of immunomodulatory drugs currently available and may represent an ideal target for therapeutic interventions. Nonetheless, treatment with CTLA4 inhibitors looked promising in isolated cases of BTCs [32]. Recent studies provide evidence for the benefit of adjuvant treatment in resected BTCs [9]. Given post-operative treatment seems to be effective in improving long-term outcome, optimisation of adjuvant therapy may be a good strategy to increase the cure rate of this otherwise deadly disease. Our data suggest that it may be worth exploring the role of immunotherapy in the adjuvant setting of BTCs as a means of preventing recurrence by re-addressing the immune response. Furthermore, immunomodulatory drugs targeting CTLA4, given post-operatively to melanoma patients, appear to provide significant benefit [33]. The BILCAP study has shown that median RFS is improved from 18 to 25 months [9]. Thus, it is likely that a subgroup of patients may get higher benefit from additional chemotherapy; thus, identification of prognostic factors that indicate the risk of relapse may aid patient selection. In our data, we observed that the expression of CTLA4 in the peritumoural area seems to have a high prognostic value, as it reflects the capacity of the host immune system to react against the tumour. Extraction of RNA from the AT may be more challenging in comparison to TT. However, modern techniques, such as nCounter analysis, enable the assessment of mRNA transcripts from small amounts of RNA obtained from formalin-fixed paraffin-embedded tissues [34]. This has already allowed gene expression profiling to guide adjuvant chemotherapy in other solid tumours such as breast cancer [35], [36].

Emerging evidence is supporting the role of Tregs in shaping tumour sensitivity to chemotherapy. Low stromal Treg density is associated with better responses to chemo-radiotherapy [10], and the depletion of circulating Treg following chemotherapy is associated with better outcomes [11]. CTLA4 is expressed on the surface of Tregs and has to bind to CD80 on antigen-presenting cells to exert inhibitory effects on cytotoxic cells [24]. Thus, it is likely that the association we have observed between strong CD80 expression and resistance to adjuvant chemotherapy may reflect the enrichment of activated Tregs in the microenvironment, which inhibit response to chemotherapy. The assessment of CD80 by IHC has two advantages: 1) allows differentiation of those cases with a strong expression compared to differentiation just in two groups and 2) is a standardised method that can be easily reproduced and taken into clinical practice, provided confirmation and validation of our findings are achieved in larger prospective cohorts.

Our study suggests that the microenvironment of BTCs is characterised by a deregulation of the immune system towards an immunosuppressive phenotype and may include activation of Tregs that confer aggressiveness and chemo-resistance. Owing to the limited number of patients in our study, definitive conclusions cannot be made. However, we believe that these observations deserve further validation in large, prospective studies to assess the role of immunomodulatory transcripts as prognostic factors in resected BTCs and support the investigation of immunomodulatory drugs in BTCs.

## Funding

Financial support: CB is a recipient of an Institute of Cancer Research Clinician Scientist Fellowship, a Marie Curie Career Integration Grant from the European Union, an Early Diagnosis Award from Pancreatic Cancer and an NIHR Royal Marsden/ICR Biomedical Research Centre project grant. NV is a recipient of a Cancer Research UK Career Development Award (A18052), a Marie Curie Career Integration Gran from the European Union, an NIHR Royal Marsden/ICR Biomedical Research Centre Flagship grant. We acknowledge Italian Association for Cancer Research (AIRC Regional grant n. 6421 to MF and MR).

## Conflict of interest statement

None declared.

## Figures and Tables

**Figure:**
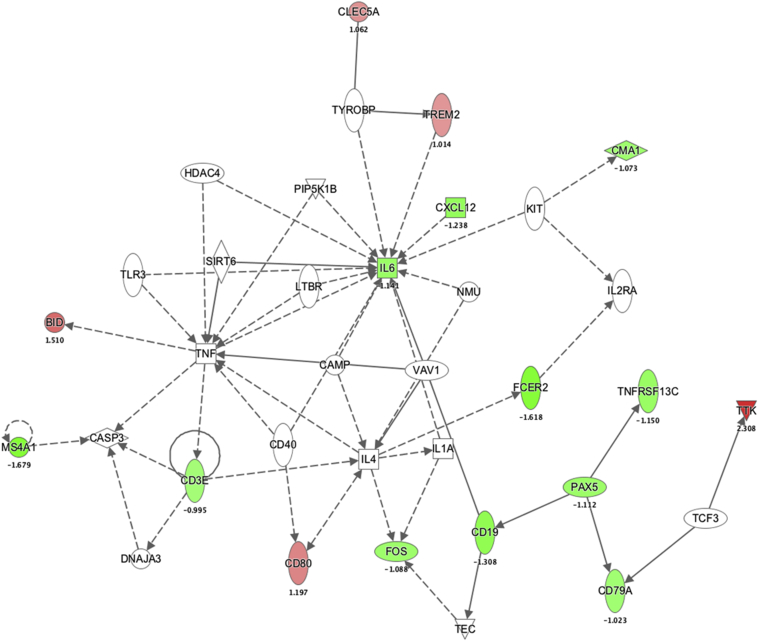


**Figure:**
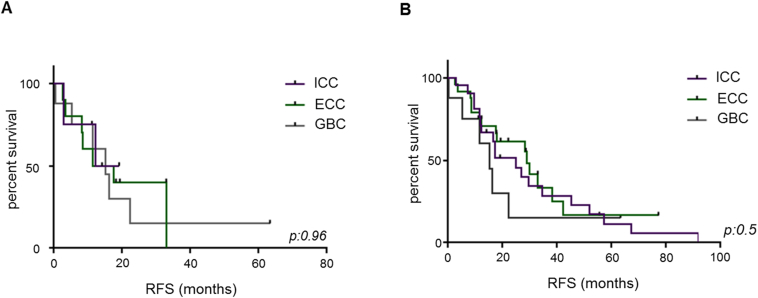


**Figure:**
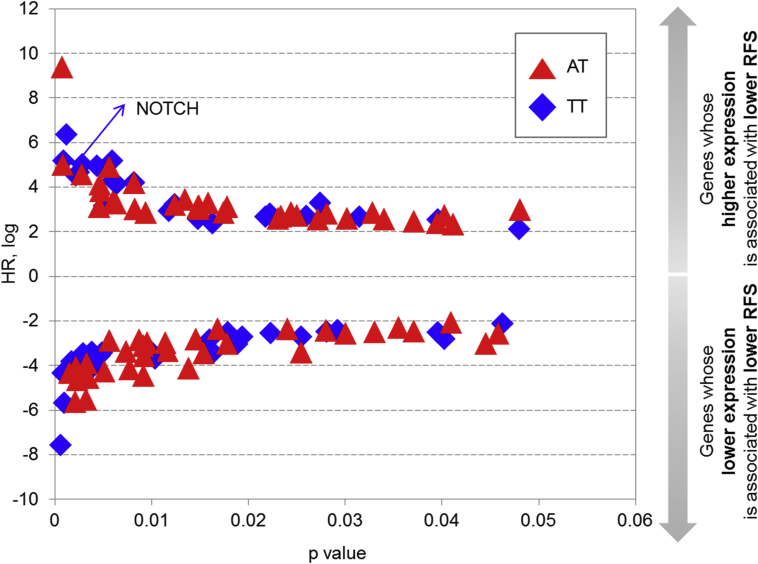


**Figure:**
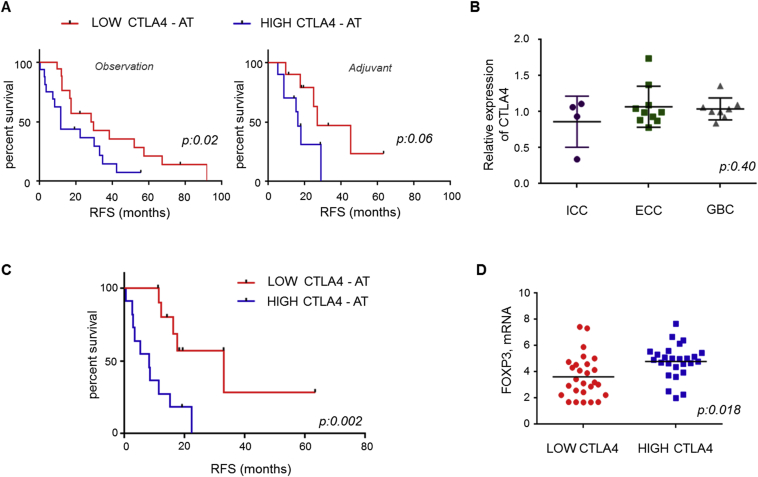


**Figure:**
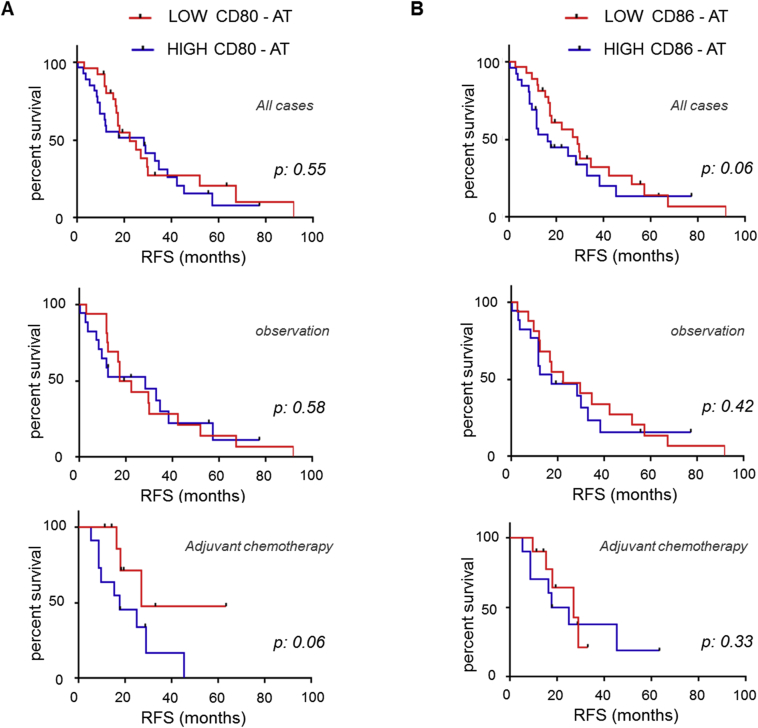


**Figure:**