# Supplementary material for: Characterisation of the immune-related transcriptome in resected biliary tract cancers
Source: Eur J Cancer. 2017 Nov;86:158–65. doi: 10.1016/j.ejca.2017.09.005 (PMC5699791; doi:10.1016/j.ejca.2017.09.005)
Supplement: Supplementary file 1 [file mmc1.docx]

**Characterization of the immune-related transcriptome in resected biliary tract cancers**

Short title: Immunoprofiling in cholangiocarcinoma

Michele Ghidini^1,2,3*^, Luciano Cascione^4*^, Pietro Carotenuto^1^, Andrea Lampis^1^, Francesco Trevisani^1,5^, Maria Chiara Previdi^1^, Jens Claus Hahne^1^, Ian Said-Huntingford^1^, Maya Raj^1^, Alessandro Zerbi^2^, Claudia Mescoli^6^, Umberto Cillo^6^, Massimo Rugge^6^, Massimo Roncalli^7^, Guido Torzilli^2,7^, Lorenza Rimassa^2^, Armando Santoro^2,7^, Nicola Valeri^1,8^, Matteo Fassan^6^, Chiara Braconi^1,8§^

^1^ The Institute of Cancer Research, London, UK

^2^ Humanitas Cancer Center, Humanitas Clinical and Research Center, Rozzano (Milan), Italy

^3^ ASST Hospital of Cremona, Cremona, IT

^4^ Institute of Oncology Research, Bellinzona, CH

^5^ San Raffaele Scientific Institute, Milan, IT

^6^ University of Padua, Padua, IT

^7^ Humanitas University, Rozzano (Milan), IT

^8^ The Royal Marsden NHS Foundation Trust, London and Surrey, UK.

^*^ These authors have contributed equally to the study.

^§^ Corresponding author: Dr Chiara Braconi, The Institute of Cancer Research, 15 Cotswold Rd, Haddow lab (room 7N1), Sutton, SM2 5NG UK, Tel: 0044 0208 722 4526, fax: 00400208 915 6634; email:chiara.braconi@icr.ac.uk

Contents of Supplementary Information

-Supplementary methods

-Supplementary Figure Legends

-Supplementary Tables and Legends

**SUPPLEMENTARY METHODS**

***RNA extraction***

Formalin fixed paraffin embedded (FFPE) tissue specimens were retrieved retrospectively. Neoplastic cellularity was assessed by microscopic examination of the original haematoxylin and eosin (H&E) slides. Tumour and adjacent tissues were separated by manually microdissecting eight consecutive 10 µm thick sections. Total RNA was extracted by using Ambion Recover All™ Total Nucleic Acid Isolation (Life Technologies™, Paisley, UK) according to the manufacturer’s instructions.

***Nanostring***

mRNA expression profiling was analysed with a commercially available nCounterPanCancer Immune Profiling from NanoString Technologies (Seattle, WA, USA), as per manufacturer’s instructions. Sample-specific background was calculated by adding the average of all the negative controls to their standard deviation multiplied by two and then subtracting this value from the raw values for each gene. Biological normalization was performed to correct for differences in sample abundances. Each sample was normalized according to the quantile normalization method, and the data were log transformed (base 2) for analysis. A first normalization was performed in the exploratory set (in both TT and AT). A second normalization was performed across the 53 AT samples of the whole set. The linear model for microarray (LIMMA) package in R was used on normalized counts to calculate statistical significances of pairwise comparisons between TT and AT samples. P-value was adjusted for multiple comparisons by Benjamini-Hochberg methods. Significance was accepted when *p* was less than *0.0*5.

In order to compare the predictive performance of our prognostic models (CTLA4 alone vs 43-gene signature), the estimated hazard ratio and the concordance probability estimate (CPE) of the models were evaluated. Hazard ratios greater than 1 indicate that patients with high predicted risk scores have poor clinical outcome. The model has strong predictive power if the CPE value is close to 1; CPE value close to 0.5 indicates that the model has poor predictive power (comparable to random prediction).We also used the Akaike’s Information Criterion, a goodness-of-fit criterion that rewards the model for higher loglikelihood score (logL) but penalizes it for each additional parameter (p) as follows: AIC = -2logL+2p. The model with the lowest AIC explains the data best.

***Taqman assay***

Taqman assays were done as previously described ([1](#_ENREF_1)) and normalized to that of RNA polymerase II subunit A (POLR2A) (Life Technologies, Paisley, UK).

***Immunohistochemistry (IHC)***

IHC for human B7-1/CD80 (R&D Systems, Minneapolis, MN, USA), was performed on the Benchmark-LT automated system from Leica Microsystems Bondmax (Leica, Wetzlar, Germany). Number of CD80 positive cells per 10 high power fields (HPF) was assessed and scored as follows: - (<10 cells), + (10-20 cells), ++ (20-100), +++ (>100).

**SUPPLEMENTARY FIGURE LEGENDS**

**Supplementary Figure 1. Network analysis of the transcripts deregulated >2 fold in BTC tumour tissues of the exploratory set.** Ingenuity Pathways Analysis (IPA) Network clustered 13 of the differentially expressed genes that are directly and indirectly interacting and relevant to cell cycle and cellular development.

**Supplementary Figure 2. Tumour site does not affect RFS.** Kaplan Meier curves have been derived by Mantel-Cox test. Median RFS is comparable across types in the exploratory set (ICC: 15.6, ECC: 14.4, GBC: 15.2 months; p:0.96) (**A**) as well as in the entire set of patients (ICC: 24.8, ECC: 28.8, GBC: 15.2 months; p:0.5) (**B**).

**Supplementary Figure 3. Multivariate analysis in the exploratory set.** nCounter PanCancer Immune Profiling was run in the Tumour Tissue (TT) and Adjacent Tissue (AT) of 22 samples of the exploratory set. Univariate analysis (UVA) was run with gene expression (high vs low, using median as cut-off) and RFS, followed by multivariate analysis (MVA). Co-variables in the MVA were gene expression (low vs high), T (T1+T2 vs T3+T4), N (N0 vs N1), site of tumour (ICC vs ECC vs GBC), resection margins (R0 vs R1), adjuvant treatment (chemotherapy vs observation). Genes which were significant at the UVA and MVA analyses are reported in the graph, where each gene is identified by Hazard Ratio (HR) (y axis) and p values (x axis). Forty-eight genes in the TT, and 71 genes in the AT were associated to RFS.

**Supplementary Figure 4. CTLA4 expression correlates with FOXP3. A.** CTLA4 mRNA expression was assessed by nCounter technologies in the AT of the entire set (n:53) and correlated to RFS in the group of patients undergoing observation alone or adjuvant chemotherapy. Separation of the curves is observed in both cases. **B**. CTLA4 expression mRNA was assessed by nCounter technologies in the TT and AT of the exploratory set (n:22). Relative expression of CTLA4 in the TT vs AT is shown. Anova test was applied for comparison of the three groups, showing no differences across the subtypes in a cohort where CTLA4 was still correlated with RFS (**C**). **D**. Data on the expression of AT CTLA4 and FOXP3 mRNA are presented (n:53). Cases with high expression of CTLA4 do shown high expression of FOXP3. Mann Whitney test (p:0.018) and ttest (p:0.005) showed statistical significance.

**Supplementary Figure 5. CD80 and CD86 mRNA expression and RFS.** CD80 (**A**) and CD86 (**B**) mRNA expression was assessed by nCounter technologies in the AT (n:53) and correlated to RFS in the group of patients undergoing observation alone or adjuvant chemotherapy. Groups were divided in low and high expression according to median. In patients receiving adjuvant chemotherapy AT median RFS was 26.87 months in cases with low AT CD80 mRNA and 19.72 months in cases with high AT CD80 mRNA expression.

**Supplementary Figure 6. Prognostic value of a gene signature derived from gene expression in the AT.** A gene signature was derived by analyzing the genes in the AT that were associated to RFS. Patients were divided into high risk or low risk according to the the gene signature. Median overall survival was 12.3 months for the high risk group and 34.5 months for the low risk group. HR 2.86 [95%CI 1.45-5.64].

**SUPPLEMENTARY TABLES AND LEGENDS**

**

**

**Supplementary Table 1. Transcripts deregulated in TT vs AT in the exploratory set.** nCounter PanCancer Immune Profiling was run in the tumour tissue (TT) and matched adjacent tissue (AT) of 22 samples of the exploratory set. Paired LIMMA analysis was performed between TT and AT, and p value adjusted for multiple comparisons using Benjamini-Hochberg correction. Reported are transcripts changed by greater than 2 fold with adjusted p values.

**

**

**Supplementary Table 2. Ingenuity pathway analysis (IPA) of the transcripts deregulated in TT vs AT.** Transcripts deregulated by >2 fold in TT vs AT were selected and used for analysis by IPA 4.0 (Ingenuity Systems Inc., [www.ingenuity.com](http://www.ingenuity.com/)). Here are reported the top classes in each category.

**

**

**Supplementary Table 3. Multivariate analysis of gene expression in the AT.** nCounter PanCancer Immune Profiling was run in the adjacent tissue (n:53). Univariate analysis (UVA) was run with gene expression (low vs high, using median as cut-off) and RFS comparing the survival curves with log-rank test, followed by multivariate analysis (MVA). Co-variables were gene expression (high vs low), T (T1 vs T2 vs T3 vs T4), N (N0 vs N1 vs Nx), site of tumour (ICC vs ECC vs GBC), resection margins (R0 vs R1), adjuvant treatment (chemotherapy vs observation), Institution. Genes which were significant at the UVA and MVA analyses are reported here along with the Hazard Ratio (HR) and p values at MVA.

|  |  |  |  |  |  |  |
| --- | --- | --- | --- | --- | --- | --- |
|  |  |  |  |  |  |  |
|  |  | **p value** | **HR** | **95% CI Low** | **95% CI High** |  |
|  | ***CTLA4 AT expression (low as reference)*** | | | | |  |
|  | CTLA4 high | 0.02 | 2.65 | 1.17 | 6.00 |  |
|  | ***Site (ICC as reference)*** | | | | |  |
|  | ECC | 0.14 | 0.54 | 0.23 | 1.22 |  |
|  | GBC | 0.60 | 0.69 | 0.18 | 2.70 |  |
|  | ***Post-operative treatment (observation as reference)*** | | | | |  |
|  | Adjuvant chemotherapy | 0.78 | 0.88 | 0.36 | 2.14 |  |
|  | ***Resection (R0 as reference)*** | | | | |  |
|  | R1 | 0.38 | 0.54 | 0.13 | 2.16 |  |
|  | ***Nodal status (N0 as reference)*** | | | | |  |
|  | N1 | 0.32 | 0.59 | 0.21 | 1.65 |  |
|  | Nx | 0.53 | 1.32 | 0.55 | 3.17 |  |
|  | ***Tumour size (T1 as reference)*** | | | | |  |
|  | T2 | 0.69 | 0.82 | 0.31 | 2.18 |  |
|  | T3 | 0.70 | 0.76 | 0.19 | 3.02 |  |
|  | T4 | 0.33 | 2.43 | 0.40 | 14.69 |  |
|  | ***Institution (Humanitas as reference)*** | | | | |  |
|  | Padova | 0.11 | 0.40 | 0.13 | 1.22 |  |

**Supplementary Table 4. Multivariate analysis of CTLA4 expression in the AT.** Multivariate analysis (MVA) was run using the co-variables listed in the table. CTLA4 expression in the adjacent tissue maintained statistical significance.

**REFERENCES**

1. Carotenuto P, Fassan M, Pandolfo R, Lampis A, Vicentini C, Cascione L, Paulus-Hock V, et al. Wnt signalling modulates transcribed-ultraconserved regions in hepatobiliary cancers. Gut 2016.
